# Intraoperative zero-heat-flux thermometry overestimates nasopharyngeal temperature by 0.39 °C: an observational study in patients undergoing congenital heart surgery

**DOI:** 10.1007/s10877-024-01204-8

**Published:** 2024-08-10

**Authors:** Ivo F. Brandes, Theodor Tirilomis, Marcus Nemeth, Johannes Wieditz, Anselm Bräuer

**Affiliations:** 1 Department of Anesthesiology, Emergency and Intensive Care Medicine, University Medical Center Göttingen, Robert-Koch-Str. 40, 37075 Göttingen, Germany; 2 Department of Cardiac, Thoracic and Vascular Surgery, University Medical Center Göttingen, Göttingen, Germany; 3 Department of Medical Statistics, University Medical Center Göttingen, Göttingen, Germany

**Keywords:** Zero-heat-flux thermometer, Hypothermia, Congenital heart disease, Core temperature, Non-invasive monitoring

## Abstract

During surgery for congenital heart disease (CHD) temperature management is crucial. Vesical (T_ves_) and nasopharyngeal (T_NPH_) temperature are usually measured. Whereas T_ves_ slowly responds to temperature changes, T_NPH_ carries the risk of bleeding. The zero-heat-flux (ZHF) temperature monitoring systems SpotOn™ (T_SpotOn_), and Tcore™ (T_core_) measure temperature non-invasively. We evaluated accuracy and precision of the non-invasive devices, and of T_ves_ compared to T_NPH_ for estimating temperature. In this prospective observational study in pediatric and adult patients accuracy and precision of T_SpotOn_, T_core_, and T_ves_ were analyzed using the Bland-Altman method. Proportion of differences (PoD) and Lin´s concordance correlation coefficient (LCC) were calculated. Data of 47 patients resulted in sets of matched measurements: 1073 for T_SpotOn_ vs. T_NPH_, 874 for T_core_ vs. T_NPH_, and 1102 for T_ves_ vs. T_NPH_. Accuracy was − 0.39 °C for T_SpotOn_, -0.09 °C for T_core_, and 0.07 °C for T_ves_. Precisison was between − 1.12 and 0.35 °C for T_SpotOn_, -0.88 to 0.71 °C for T_core_, and − 1.90 to 2.05 °C for T_ves_. PoD ≤ 0.5 °C were 71% for T_SpotOn_, 71% for T_core_, and 60% for T_ves_. LCC was 0.9455 for T_SpotOn_, 0.9510 for T_core_, and 0.9322 for T_ves_. Temperatures below 25.2 °C (T_SpotOn_) or 27.1 (T_core_) could not be recorded non-invasively, but only with T_ves_. Trial registration German Clinical Trials Register, DRKS00010720.

## Clinical implications of the study

**What is already known about the topic?:**

In order to maintain normothermia perioperatively, measuring body core temperature is crucial. New temperature measurement systems are able to non-invasively obtain body core temperature in adults and in children in non-cardiac surgery as well as in adults in cardiac surgery.

**What new information does this study add?:**

To our knowledge this is the first study in paediatric patients undergoing surgery for congenital heart disease to compare two different non-invasive temperature management systems to standard of care nasopharyngeal and vesical temperature measurement.

## Background

Patients undergoing surgery for congenital heart disease (CHD) are often subjected to large temperature changes on cardiopulmonary bypass (CPB). In addition, during procedures without CPB unintended perioperative hypothermia defined as core temperature < 36.0 °C occurs frequently [1]. Many well-conducted prospective, randomized trials [2–5], and large retrospective studies [6, 7] documented numerous adverse events associated with hypothermia, like increased blood loss [4, 8, 9], higher amount of perioperative transfusions [2, 4, 10–12], and surgical site infections [2, 3, 13, 14]. An essential part of thermal management strategy is the reliable measurement of core temperature. The ideal temperature measurement method should provide reliable, reproducible values safely and conveniently [15], using a small, non-invasive, and easy to use device [16]. In the last years, several non-invasive devices became available, and have been evaluated in several studies. However, there is a lack of data regarding patients undergoing surgery for CHD. This group of patients differs from patients for surgery for non-CHD, because they cover a broader age range, and the temperature range used in surgery for CHD is wider than in surgery for non-CHD.

In many institutions vesical temperature (T_ves_) is the standard of care during and especially after cardiac surgery because it provides continuous readings, stays securely in place even during positioning of the patient, and gives stable measurements regardless of urine flow rate [15]. But as vesical temperature is only slowly adapting to rapid temperature changes [17], a nasopharyngeal temperature probe is used additionally. It also yields reliable, accurate values continuously, but carries the risk of accidental dislocation and nasal bleeding [18]. Most patients undergoing surgery for CHD need CPB and are fully heparinized. Therefore, any procedure with a risk of bleeding should be avoided. Gold standard methods of core temperature measurement like pulmonary artery temperature are available for children, but do not cover all age ranges. Esophageal temperature is not sensible during intrathoracic procedures because of the proximity to the operating field. Therefore, an accurate and reliable non-invasive monitor would be helpful. Unfortunately, many of these methods are not very reliable [19]. A better alternative could be a cutaneous sensor based on heat flux technology. The SpotOn™ zero-heat-flux (T_SpotOn_) thermometer and the Tcore™ (T_core_) thermometer are two devices that measure body core temperature non-invasively. Some studies could show that these devices could continuously measure body core temperature with an acceptable accuracy and precision via a forehead sensor in surgical patients in comparison with an accepted reference method [20–25]. However, there is a lack of data regarding patients undergoing surgery for CHD.

Therefore, we evaluated the measurement characteristics of these sensors, including accuracy and precision of T_SpotOn_, T_core_, and vesical temperature (T_ves_) compared to the standard nasopharyngeal temperature (T_NPH_) in patients undergoing surgery for CHD.

## Materials and methods

In this study two non-invasive devices with slightly different measuring techniques have been used to estimate body core temperature. Both devices estimate core temperature from the surface of the skin, being placed typically on the lateral forehead of the patient. The temperature of uninsulated skin depends greatly on ambient conditions. If a small area of the skin could be fully insulated, the surface temperature would eventually closely approximate the tissue temperature directly beneath it. The SpotOn™ Temperature Monitoring System (T_SpotOn_) is a direct-contact zero-heat-flux thermometer. It creates such a zone of perfect insulation by heating the skin beneath the sensor, and by insulating the skin at the same time [26, 27]. Consisting of a self-adhesive single use probe and a controller, the self-adhesive probe is actively heated by the controller, thereby creating a small isothermal zone of tissue in which almost no heat transfer to the environment occurs [28]. The T_core_ system is also a zero-heat-flux system, but without a heating device and a controller. It features two temperature probes on each side of a standardized insulator with a known heat transfer coefficient [24, 29], both incorporated in a self-adhesive single use probe.

This prospective single-center observational study was conducted at the University Medical Centre Göttingen, Germany, after obtaining local ethics committee approval (No. 5/3/16), and registration on the German Clinical Trials Register (DRKS00010720). We followed STROBE guidelines for reporting of observational studies [30].

Inclusion criteria were patients aged 0 to 60 years undergoing surgery for congenital heart disease, ASA status II-IV. Exclusion criteria were refusal to participate, ASA status V, preoperative fever, a clinically relevant thyroid disease, pregnancy, known allergy to flunitrazepam, midazolam, sufentanil, rocuronium, or sevoflurane, or participation in another clinical trial.

Written informed consent was obtained from all patients, parents, or legal guardians at least on the day prior to anesthesia and surgery. Patients underwent elective surgery for congenital heart disease with or without cardiopulmonary bypass (CPB) with balanced anaesthesia using midazolam, sufentanil, rocuronium, and sevoflurane.

After arrival in the OR, patients were prepared for surgery. The sensors of the non-invasive devices SpotOn™ (3 M™ Health Care, St. Paul, MN, U.S.A.; meanwhile distributed under the product name 3 M™ Bair Hugger™ Temperature Monitoring System) and Tcore™ (Drägerwerk AG & Co. KGaA, Lübeck, Germany) were applied to the forehead before induction of anesthesia and were removed at the end of the surgery. After a short time of equilibration, both devices provided stable readings of the body core temperature. After induction of anesthesia, a bladder catheter (Teleflex Rüsch Sensor 400, Teleflex Medical Sdn. Bhd., Kamunting, Malaysia) was established, and a reusable temperature probe was placed deep into the nasopharynx (Philips 21,075 A, Philips Medical Systems, Andover, MA, U.S.A.), to obtain vesical and nasopharyngeal temperature readings, respectively. Intraoperatively body core temperature was measured with both non-invasive devices, the vesical and the nasopharyngeal probe before, during, and after cardiopulmonary bypass (CPB) every 15 min until the end of surgery. During CPB, the blood temperature in the arterial branch of the oxygenator (Terumo Capiox, Terumo Cardiovascular Systems, Ann Arbor, MI, U.S.A.) was additionally obtained.

For each patient the following data were documented: age, height, weight, sex, ASA-classification, duration of surgery, bypass- and cross clamp time.

We used the method described by Bland-Altman for comparison of differences with multiple measurements, adjusted for unequal numbers of measurements per patient [31], where accuracy (bias = mean difference between methods) and precision (limits of agreement = 1.96 standard deviations) are used for interpretation of data. Further, we calculated the proportion of all differences that were within a predefined threshold of ± 0.5 °C [32], and Lin’s concordance correlation coefficient to assess the agreement between pairs of observations [33].

A sample size of 50 patients with congenital heart disease was considered sufficient to demonstrate a clinically meaningful difference, as there are no formal rules for power calculations for this method. Data were analysed with MedCalc^®^ Statistical Software version 19.8 (MedCalc Software Ltd, Ostend, Belgium; https://www.medcalc.org; 2021).

We also recorded the minimal and maximal temperature measured with each device. In addition, we looked for any lesions from the non-invasive temperature sensors.

## Results

**Fig. 1:**
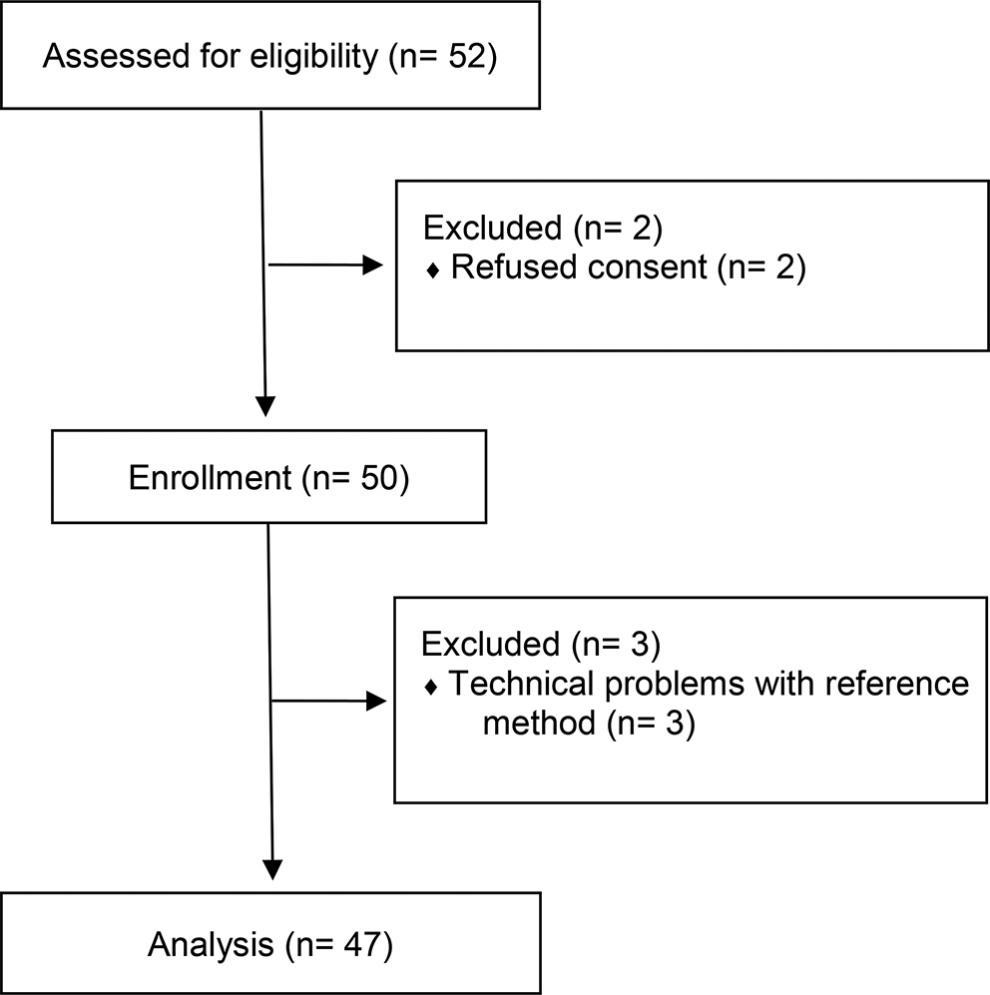
Flowchart of enrollment of patients

After assessing 52 patients for eligibility, 50 patients (26 male/24 female) could be enrolled in this study between July 2016 and May 2017. One patient needed a second procedure and was included again after informed consent, so there are two sets of measurement from this patient. Of these 51 sets of measurements, data from 3 patients had to be excluded due to technical problems with the reference method (see Fig. 1).

The remaining 47 patients were 2 days to 57 years of age and had a median (1st -3rd quartile) age of 4.5 years (0.8–18 y). The patients´ characteristics are presented in Table 1.

**Table 1:** Characteristics of enrolled patients.

	Median	1st quartile	3rd quartile	Min	Max
**Age [years]**	4.5	0.8	18	0.0	57.0
**Height [cm]**	102	63	164	50.0	181.0
**Weight [kg]**	15	5.9	58	3.2	127.0
**Surgery [min]**	273	218	364	123	1218
**X-Clamp [min]**	72	56	110	14	203
**CPB [min]**	142	101	202	39	853
**ASA-Status**	**n**	**Male**	**Female**		
**II**	12	26	22		
**III**	34				
**IV**	2				
		**n**	**%**		
**Age [years]**	< 1	21	43.8		
	≥ 1 and < 2	3	6.3		
	≥ 4 and < 5	4	8.3		
	≥ 5 and < 6	1	2.1		
	≥ 6 and < 7	2	4.2		
	≥ 9 and < 18	6	12.5		
	> 18	11	22.9		
	≤ 2	24	50		
	≥ 2	24	50		

X-Clamp = cross clamp time of the aorta; CPB = cardiopulmonary bypass; ASA = American Society of Anesthesiologists

In all patients the SpotOn^TM^-sensor was used, but due to supply shortcomings, 8 of 47 patients were not monitored with the Tcore^TM^-sensor. In 3 patients CPB was not used, and in 3 patients cross clamping of the aorta was not necessary. Data from these patients resulted in 1073 sets of matched T_SpotOn_and T_NPH_ measurements, 874 sets of matched T_core_ and T_NPH_ measurements, and 1102 sets of matched T_ves_ and T_NPH_ measurements.

Each device has a different range of temperature that was measured (s. Table 2).

**Table 2:** Range of temperature measured in this study with different devices.

	T_SpotOn_	Tcore	Vesical	NPH	CPB
Min temperature [°C]	25.2	27.1	18.0	16.8	16.5
Max temperature [°C]	39.2	39.6	38.9	38.9	37.4

NPH = nasopharyngeal; CPB = cardiopulmonary bypass; Min = minimal; Max = maximal

**Fig. 2:**
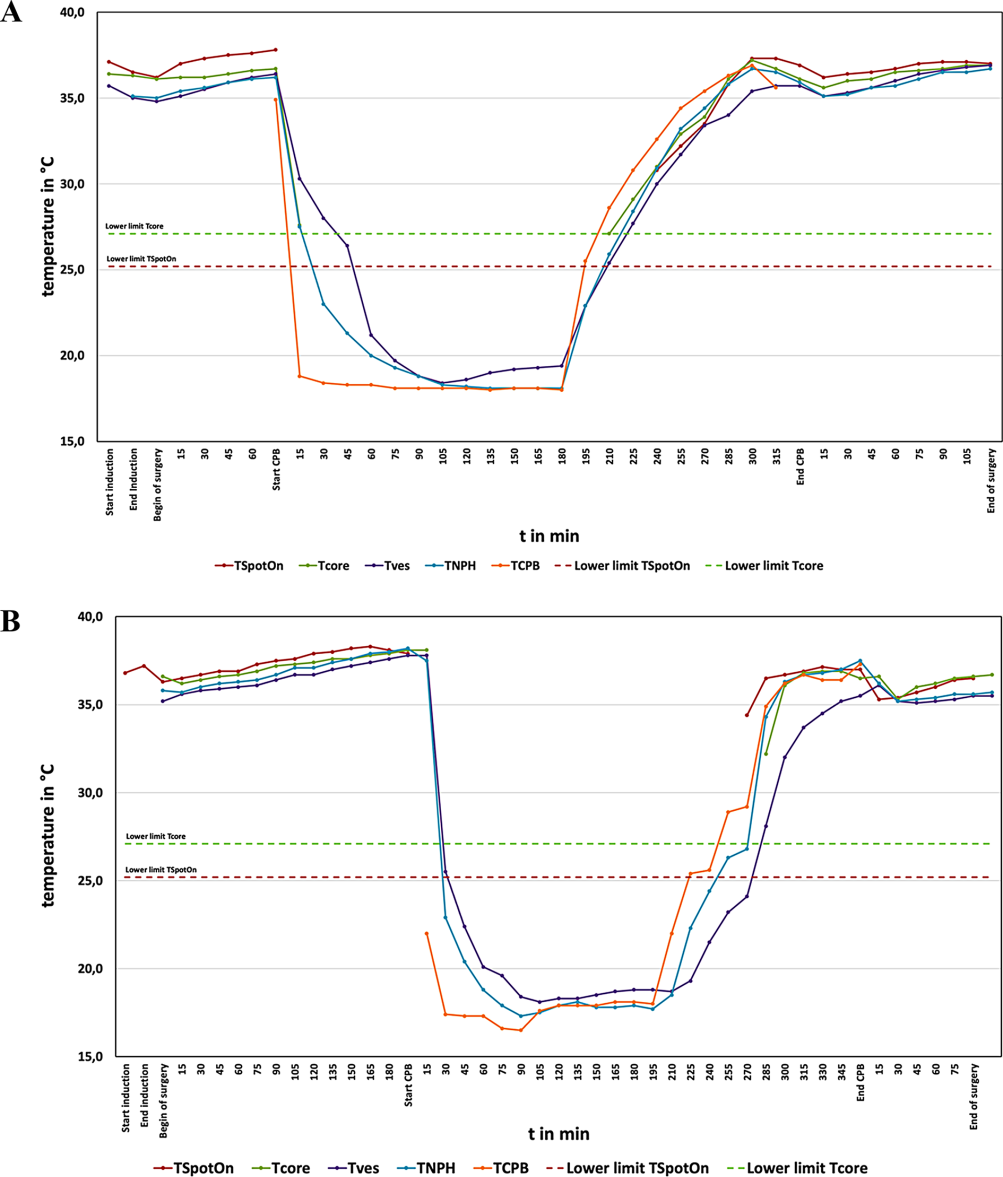
(**a**) Temperatures measured with SpotOn™ (T_SpotOn_), Tcore™ (T_core_), vesical (T_ves_) and nasopharyngeal (T_NPH_) temperature probes in a 2-day-old patient. During cardiopulmonary bypass temperature was measured in the arterial branch of the oxygenator (T_CPB_). (**b**) Temperatures measured with SpotOn™ (T_SpotOn_), Tcore™ (T_core_), vesical (T_ves_) and nasopharyngeal (T_NPH_) temperature probes in a 15-year-old patient. During cardiopulmonary bypass temperature was measured in the arterial branch of the oxygenator (T_CPB_)

Due to the thermistors used in the non-invasive devices, temperatures below 25.2 °C (T_SpotOn_) or 27.1 °C (T_core_) could not be measured. No limitations were discovered with vesical or nasopharyngeal temperature measurements when compared to temperature on CPB. Figure 2a and b show typical examples of the measured temperature with the different devices. From the figures it is obvious that fast changes in body temperature were not simultaneously detected by all temperature measurement devices used. To further analyze these differences, more data points than every 15 min would be necessary.

### Bland-altman analysis

**Fig. 3:**
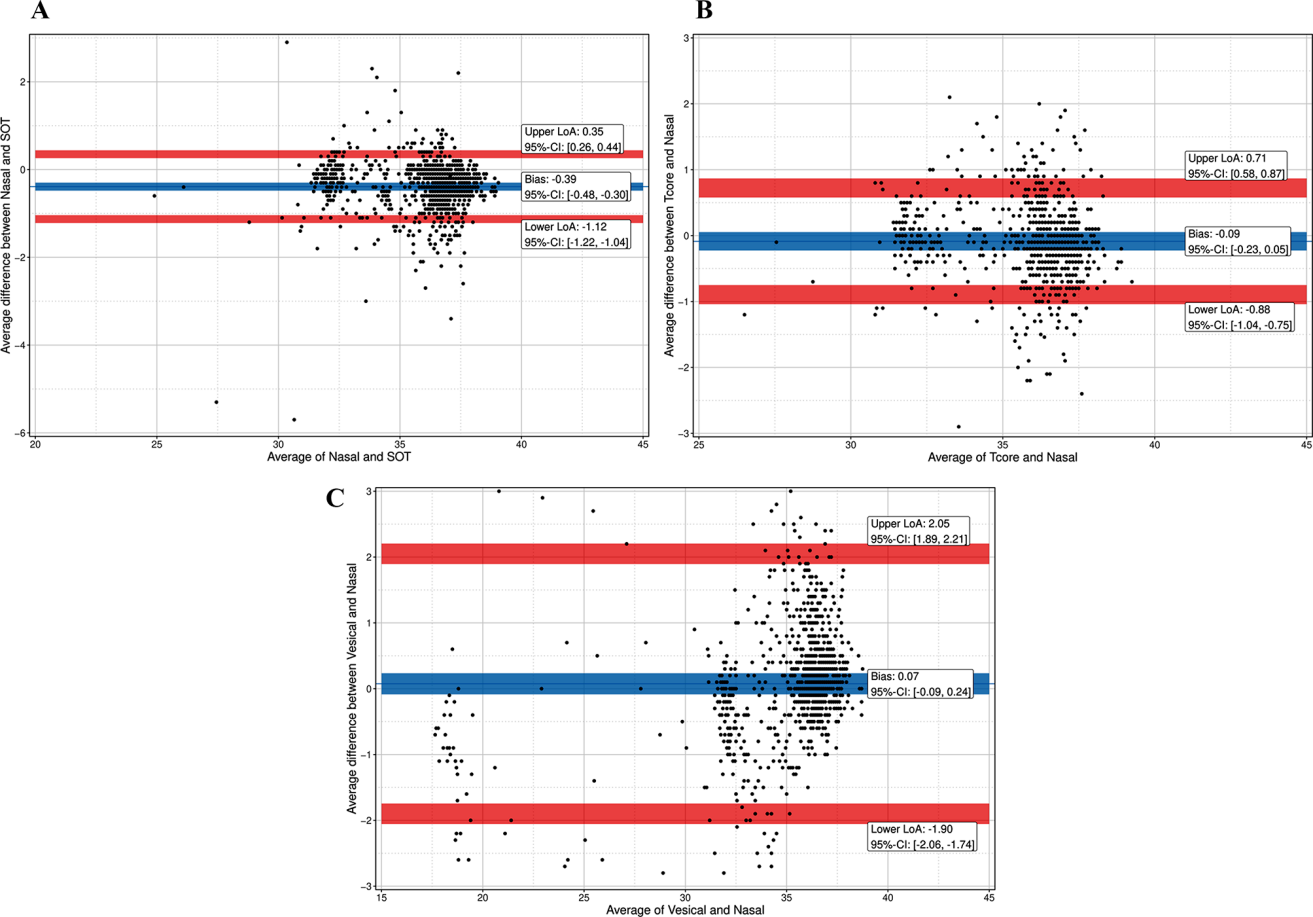
(**a**) Bland-Altman plot with multiple temperature measurements (47 patients with 1073 measurement pairs) of the SpotOn^TM^-Sensor (SOT) and a nasopharyngeal probe (Nasal). Blue line indicates mean bias with 95% confidence interval (CI): -0.39 °C (-0.48; -0.30); red lines indicate 95% limits of agreement (LoA) with 95% confidence interval (CI). LoA: ±0.74 °C, lower LoA: -1.12 °C (-1.22; -1.04), upper LoA: 0.35 °C (0.26; 0.44). (**b**) Bland-Altman plot with multiple temperature measurements (39 patients with 874 measurement pairs) of the Tcore^TM^-Sensor (T_core_) and a nasopharyngeal probe (T_NPH_). Blue line indicates mean bias with 95% confidence interval (CI): -0.09 °C (-0.23; 0.05); red lines indicate 95% limits of agreement (LoA) with 95% confidence interval (CI). LoA: ±0.80 °C, lower LoA: -0.88 °C (-1.04; -0.75), upper LoA: 0.71 °C (0.58; 0.87). (**c**) Bland-Altman plot with multiple temperature measurements (47 patients with 1102 measurement pairs) of a vesical (T_ves_) and a nasopharyngeal probe (T_NPH_). Blue line indicates mean bias with 95% confidence interval (CI): 0.07 °C (0.09; 0.24); red lines indicate 95% limits of agreement (LoA) with 95% confidence interval (CI). LoA: ±1.98 °C, lower LoA: -1.90 °C (-2.06; -1.74), upper LoA: 2.05 °C (1.89; 2.21)

For all patients together, bias was − 0.39 °C for T_SpotOn_versus T_NPH_, -0.09 °C for T_core_ versus T_NPH_, and 0.07 °C for T_ves_ versus T_NPH_. For LoA and CI see Fig. 3a-c; Table 3.

**Fig. 4:**
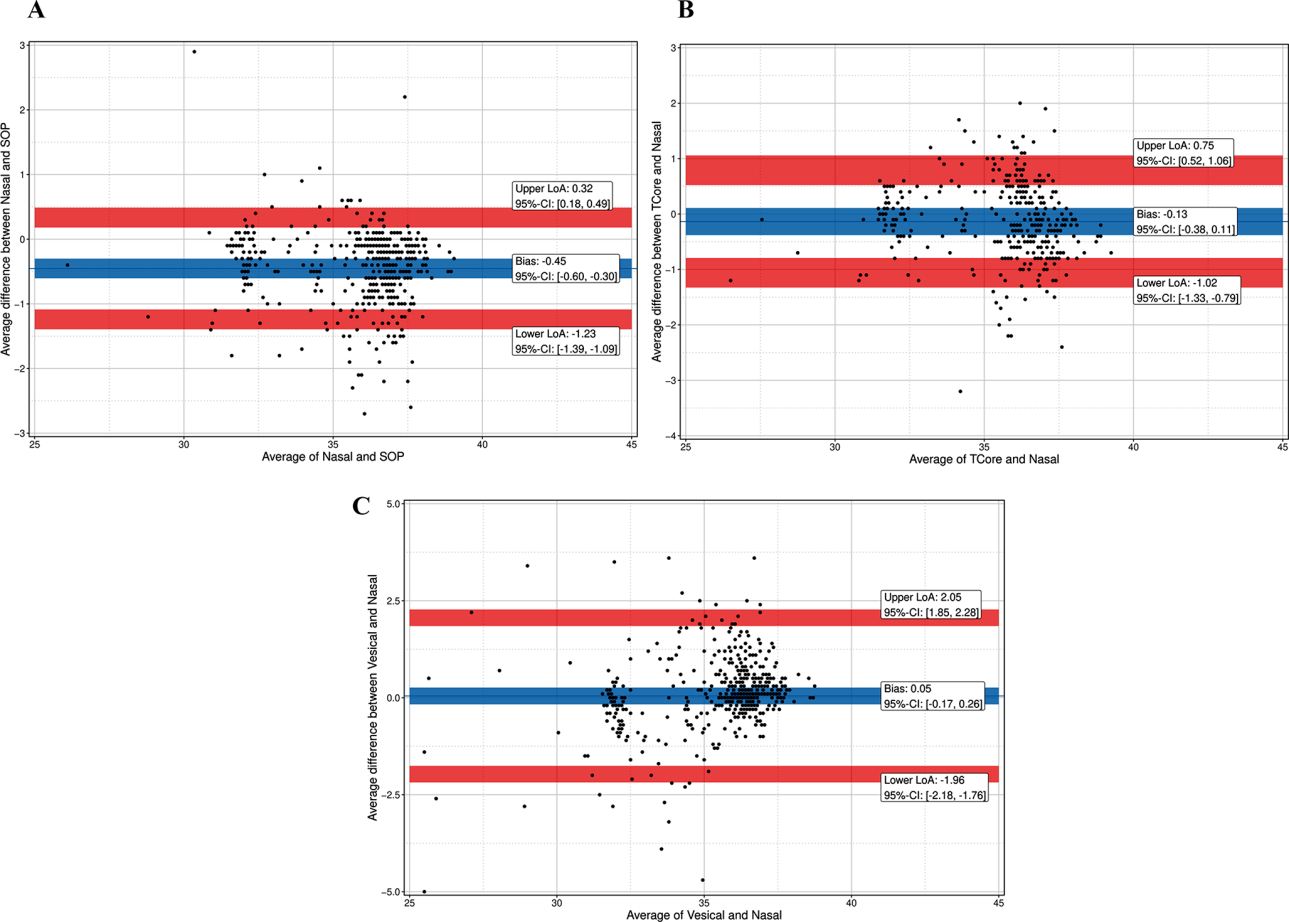
(**a**) Bland-Altman plot with multiple temperature measurements of the SpotOn^TM^-Sensor (SOT) and a nasopharyngeal probe (Nasal). Blue line indicates mean bias with 95% confidence interval (CI): -0.45 °C (-0.60; -0.30); red lines indicate 95% limits of agreement (LoA) with 95% confidence interval (CI). LoA: ±0.78 °C, lower LoA: -1.23 °C (-1.39; -1.09), upper LoA: 0.32 °C (0.18; 0.49). (**b**) Bland-Altman plot with multiple temperature measurements of the Tcore^TM^-Sensor (T_core_) and a nasopharyngeal probe (T_NPH_). Blue line indicates mean bias with 95% confidence interval (CI): -0.13 °C (-0.38; 0.11); red lines indicate 95% limits of agreement (LoA) with 95% confidence interval (CI). LoA: ±0.89 °C, lower LoA: -1.02 °C (-1.33; -0.79), upper LoA: 0.75 °C (1.06; 0.52). (**c**) Bland-Altman plot with multiple temperature measurements of a vesical (T_ves_) and a nasopharyngeal probe (T_NPH_). Blue line indicates mean bias with 95% confidence interval (CI): 0.05 °C (-017; 0.26); red lines indicate 95% limits of agreement (LoA) with 95% confidence interval (CI). LoA: ±2.00 °C, lower LoA: -1.96 °C (-2.18; -1.76), upper LoA: 2.05 °C (1.85; 2.28)

Stratification of the data by age class for patients younger or older than 2 years yielded the following results. For all patients age 2 years or younger, bias was − 0.45 °C for T_SpotOn_versus T_NPH_, -0.13 °C for T_core_ versus T_NPH_, and 0.05 °C for T_ves_ versus T_NPH_. For LoA and CI see Fig. 4a-c; Table 3.

**Fig. 5:**
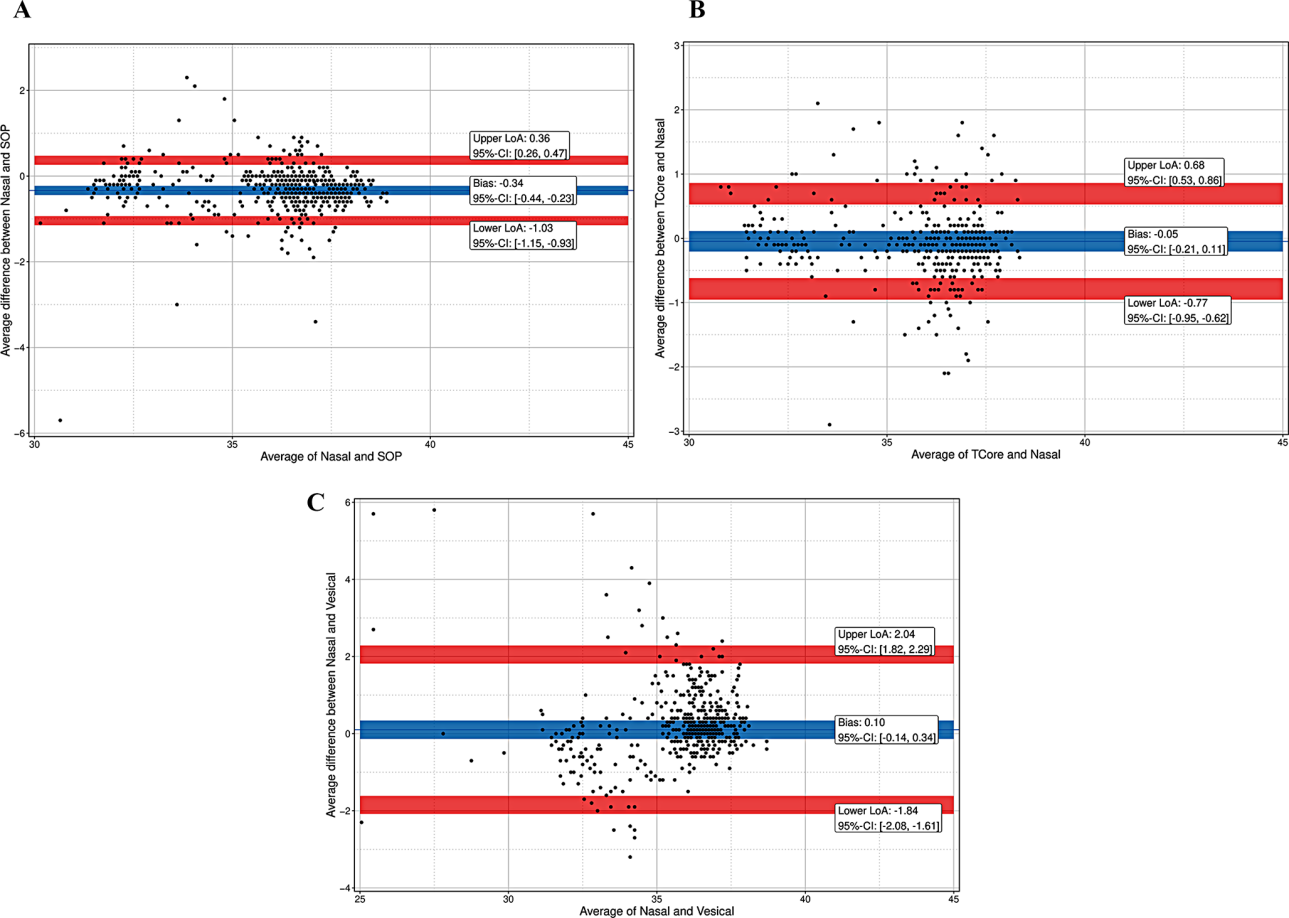
(**a**) Bland-Altman plot with multiple temperature measurements of the SpotOn^TM^-Sensor (SOT) and a nasopharyngeal probe (Nasal). Blue line indicates mean bias with 95% confidence interval (CI): -0.34 °C (-0.44; -0.23); red lines indicate 95% limits of agreement (LoA) with 95% confidence interval (CI). LoA: ±0.70 °C, lower LoA: -1.03 °C (-1.15; -0.93), upper LoA: 0.36 °C (0.26; 0.47). (**b**) Bland-Altman plot with multiple temperature measurements of the Tcore^TM^-Sensor (T_core_) and a nasopharyngeal probe (T_NPH_). Blue line indicates mean bias with 95% confidence interval (CI): -0.05 °C (-0.21; 0.11); red lines indicate 95% limits of agreement (LoA) with 95% confidence interval (CI). LoA: ±0.73 °C, lower LoA: -0.77 °C (-0.95; -0.62), upper LoA: 0.68 °C (0.53; 0.86). (**c**) Bland-Altman plot with multiple temperature measurements of a vesical (T_ves_) and a nasopharyngeal probe (T_NPH_). Blue line indicates mean bias with 95% confidence interval (CI): 0.10 °C (-0.14; 0.34); red lines indicate 95% limits of agreement (LoA) with 95% confidence interval (CI). LoA: ±1.94 °C, lower LoA: -1.84 °C (-2.08; -1.61), upper LoA: 2.04 °C (1.82; 2.29)

For all patients of age older than 2 years, bias was − 0.34 °C for T_SpotOn_versus T_NPH_, -0.05 °C for T_core_ versus T_NPH_, and 0.10 °C for T_ves_ versus T_NPH_. For LoA and CI see Fig. 5a-c; Table 3.

**Table 3:** Bias and limits of agreement for all measurement devices stratified by age.

Age	NPH vs. T_SpotOn_		NPH vs. T_core_		T_ves_ vs. NPH
all	-0.39 ± 0.74		-0.09 ± 0.80		0.07 ± 1.98
< 2a	-0.45 ± 0.78		-0.13 ± 0.89		0.05 ± 2.00
> 2a	-0.34 ± 0.70		-0.05 ± 0.73		0.10 ± 1.94

NPH = nasopharyngeal

No clinically relevant differences between age stratification in terms of bias and limits of agreement were found.

Of the differences between T_SpotOn_ and T_NPH_, 71% were ≤ 0.5 °C, 71% of the differences between T_core_ and T_NPH_ were ≤ 0.5 °C, and 60% of the differences between T_ves_ and T_NPH_ were ≤ 0.5 °C. Lin´s concordance correlation coefficient was 0.9455 for T_SpotOn_, 0.9510 for T_core_, and 0.9322 for T_ves_.

In none of the patients we could find any burns or significant cutaneous reactions from the non-invasive cutaneous temperature sensors, nor was any bleeding noted using the nasopharyngeal probe.

## Discussion

### Bias

Compared to the standard nasopharyngeal temperature monitoring, the SpotOn™ Temperature Monitoring System (T_SpotOn_) overestimated the temperature by 0.39 °C (95%CI -0.48 to -0.30), the Tcore™ Temperature Monitoring System (T_core_) by 0.09 °C (95%CI -0.23 to 0.05), and the vesical temperature measurement underestimated it by 0.07 °C (95%CI -0.09 to 0.24) for all patients. Age stratification did not yield significant differences.

For T_SpotOn_our bias was higher compared to Bräuer et al. [34], who studied different temperature measurement methods in critically ill patients and compared the SpotOn^TM^-System with blood temperature. In a meta-analysis, Conway et al. [35] found a small bias of 0.03, but they pooled patients undergoing cardiac and non-cardiac surgery, including studies with different reference methods, e.g., esophageal, rectal, or blood temperature measurement. Hart et al. [36] found a bias of 0.3 in an emergency department setting, and Zaballos et al. [37] found a bias of 0.35 between the SpotOn^TM^-System and an esophageal probe in patients undergoing non-cardiac surgery. In children up to the age of 6 years undergoing non-cardiac surgery, Nemeth et al. reported a bias of 0.26 between the SpotOn^TM^-System and an esophageal probe [38]. In adult cardiac patients Eshraghi et al. [20] and Gómez-Romero et al. [39] found a bias of -0.23 and 0.21, respectively, between the SpotOn^TM^-System and blood temperature in the pulmonary artery. We used a nasopharyngeal probe as standard method which might have contributed to the larger bias. Boisson et al. [21] showed that rapid changes in body core temperature induced by hyperthermic intraperitoneal chemotherapy markedly increased the bias. Another reason for the bigger bias seen in cardiac surgical patients could be the rapid cooling and rewarming during cardiopulmonary bypass. The slightly higher bias in our study might be due to the use of a nasopharyngeal temperature probe as the standard temperature measurement method.

Using the Tcore^TM^-System in patients undergoing non-cardiac surgery, Kimberger et al. reported a bias of -0.08 and − 0.01, respectively, compared to temperature measured in the distal esophagus [24, 25]. Soehle et al. [40] found a bias of -0.02 in patients undergoing non-cardiac surgery between the Tcore^TM^-System and blood temperature. The bias of 0.09 in our cardiac population was higher than in the non-cardiac patient population, but Sastre et al. [41] and Gómez-Romero et al. [39] reported an even bigger bias, -0.2 and 0.48, respectively, in their adult patients undergoing cardiac surgery. Our results for the bias of the Tcore^TM^-System are of the same magnitude as reported by others.

The bias between vesical temperature and nasopharyngeal temperature was within the same range as reported by other studies [17, 42, 43].

### Limits of agreement (LoA)

The LoA were − 1.12 to 0.35 for T_SpotOn_, -0.88 to 0.71 for T_core_ and − 1.90 to 2.05 for T_ves_.

The SpotOn^TM^-System was used in several studies with diverging results. In adult patients undergoing non-cardiac surgery, West et al. [44] reported LoA of -0.71 to 0.58 compared to a nasopharyngeal temperature probe, in children in non-cardiac surgery Nemeth et al. [38] found LoA of -0.11 to 0.62 compared to a esophageal temperature probe. In ICU patients Bräuer et al. [34] reported LoA of -0.76 to 0.51 compared to blood temperature. But in cardiac surgery, the LoA were bigger. Eshraghi et al. [20] found LoA of -1.06 to 0.60 compared to the temperature in the pulmonary artery. And in a study by Gómez-Romero et al. [39] the LoA were − 2.27 to 2.71 between the SpotOn^TM^-system and pulmonary artery temperature. Conway´s metaanalysis revealed LoA of -0.93 to 0.98. It appears that in cardiac surgery, where large temperature gradients occur during cooling and rewarming, the LoA are bigger than in non-cardiac surgery. Large temperature gradients also occur during hyperthermic intraperitoneal chemotherapy and lead to bigger LoA in adult patients undergoing non-cardiac surgery [21]. These large temperature gradients between different measurements sites occur during rapid cooling and rewarming [45] and they represent differences in perfusion to different measurement sites [46]. Our findings for the LoA of the SpotOn^TM^-System fit well into the literature.

The Tcore^TM^-System was also used in several studies, both in cardiac and non-cardiac surgery. In non-cardiac surgery, Kimberger et al. [24, 25] and Soehle et al. [40] reported small LoA comparing temperature measured with the Tcore^TM^-system to esophageal temperature. Whereas in cardiac surgery Sastre et al. [41] found LoA of -1,30 to 0.80 between the Tcore^TM^-system and esophageal temperature, Gómez-Romero et al. [39] reported LoA of -2.02 to 2.98, comparing the Tcore^TM^-system to pulmonary artery temperature. Our results for the LoA of the Tcore^TM^-System are of the same magnitude as reported in other studies.

One reason why the LoA between vesical and nasopharyngeal temperature were so wide, could be the fast changes in temperature occurring during rapid cooling and rewarming on CPB. As shown above (Fig. 2a and b), the vesical temperature responds slower than the nasopharyngeal to temperature changes, especially during cooling.

### Values within ± 0.5 °C of the nasopharyngeal temperature

Only 71% of the T_SpotOn_ and T_core_ values and only 60% of the T_ves_ values were within ± 0.5 °C of nasopharyngeal temperature. Eshraghi et al. found similar values [20] in adult patients undergoing cardiac surgery using a ZHF device, compared to pulmonary artery temperature as gold standard. Other studies found higher percentages comparing ZHF devices to a standard temperature monitoring site [21, 38, 47]. But Boisson et al. used a distal esophageal or radial artery probe as body core temperature measurement site in adult non-cardiac surgery during slow temperature changes. During rapid temperature changes, these values dropped to below 40%. Nemeth et al. [38] and Carvalho et al. [47] used an esophageal and not a nasopharyngeal temperature probe in pediatric patients.

For Tcore, Kimberger et al. found 98% and 90% of their values within ± 0.5 °C of an esophageal temperature probe in adult non-cardiac surgery [24, 25], whereas Sastre et al. [41] only found 69% within ± 0.5 °C a nasopharyngeal temperature, and only 55% within ± 0.5 °C a pulmonary artery temperature in adult patients undergoing cardiac surgery. Our results support the conclusion that the Tcore^TM^-system might not be an ideal non-invasive temperature measurement system in this population.

### Lin´s concordance correlation coefficient

Lin´s concordance correlation coefficient was very high for all three measurements sites: 0.9455 for T_SpotOn_, 0.951 for T_core_, and 0.9322 for T_ves_. But because the body core temperature measured over all patients had a minimum of 16.8 °C, and a maximum of 38.9 °C, correlation tends to be very high and has to be interpreted with caution.

## Temperatures measured

Both non invasive devices were unable to measure temperatures below 25 °C. For most procedures, measuring temperatures that low is not necessary, but for some it is mandatory, e.g. if deep hypothermic cardiac arrest (DHCA) is needed for the repair of the congenital defect. Thus, those devices cannot replace nasal temperature measurement for a procedure if DHCA is needed.

### Limitations of the study

When evaluating new measurement methods, it is important to compare the results to the gold standard. The blood temperature in the pulmonary [20, 22] or iliac [21] artery is described as the most suitable reference method. As recommended by others [32, 48], we used nasopharyngeal temperature as a reference method. Also, not for all our patients a pulmonary or an iliac catheter were available.

Another potential limitation of the study is that the nasopharyngeal probe is at risk for false readings due to accidental dislocation of the probe. The use of an esophageal probe as used by Carvalho et al. [47] might also have led to false measurements because of the proximity of the operating field to the measurement site that is exposed during intrathoracic procedures. Also, the use of an esophageal temperature probe would interfere with the transesophageal echo probe, which is mandatory in most cases.

A further limitation is that we used a 15 min interval to measure temperatures. Because vesical and nasopharyngeal temperatures adapt differently to temperature changes compared to temperature measured with T_SpotOn_ and T_core_, shorter intervals might have yielded slightly different results.

In some patients, noradrenaline was used as a vasoconstrictor, which might have changed cutaneous blood flow and therefore interfere with skin temperature measurements. However, we did not document the times when a vasoconstrictor was given. In addition, we applied forced air-warming using an underbody blanket throughout the procedure, which also could have interfered with temperature measurement at the forehead. Another poorly controlled factor was the operating room temperature, which was set to 20 °C. However, we did not measure it, so we cannot rule out any effect of the ambient temperature in this respect, but we think that the warmth of the forced-air warming is probably more important in this respect.

## Conclusion

In patients undergoing surgery for congenital heart disease, temperatures obtained with a zero-heat-flux system differed slightly from a nasopharyngeal probe. The non-invasive SpotOn™ temperature monitoring system showed a mean bias of -0.39 °C, and the non-invasive Tcore™ temperature monitoring system showed a bias of -0.09 °C. Age stratification did not yield clinically relevant different results. The risk of perioperative hypothermia may be underestimated, while the risk of hyperthermia may be overestimated. Because temperatures below 25 °C could not be detected by neither non-invasive device, they are unsuitable if temperatures below 25 °C are mandatory for the repair of the congenital defect.

## Data Availability

No datasets were generated or analysed during the current study.
